# Post-infectious Myocarditis: A Rare Complication of Campylobacter Gastroenteritis

**DOI:** 10.7759/cureus.99521

**Published:** 2025-12-18

**Authors:** Tarek Al Smadi, Pia Zaldua, Aung Hein

**Affiliations:** 1 Internal Medicine, Royal Bournemouth Hospital, Bournemouth, GBR; 2 Cardiology, Royal Bournemouth Hospital, Bournemouth, GBR

**Keywords:** campylobacter gastroenteritis, campylobacter species, heart failure, myocarditis, myocarditis complication

## Abstract

Acute myocarditis involves inflammatory injury to the myocardium and may develop in association with diverse infectious or non-infectious triggers. *Campylobacter jejuni *(*C. jejuni*), a leading cause of bacterial gastroenteritis worldwide, is rarely associated with extraintestinal cardiac manifestations. This report describes a case of *C. jejuni*-associated myocarditis in a 19-year-old male patient who presented with severe central chest pain, diaphoresis, and fever three days following an episode of acute gastroenteritis. Initial investigations revealed significantly elevated cardiac biomarkers and preserved renal function. Stool polymerase chain reaction confirmed *C. jejuni* infection. Transthoracic echocardiography demonstrated a reduction in left ventricular (LV) systolic function with an ejection fraction (EF) of 43% and global hypokinesis. Cardiac magnetic resonance imaging (MRI) confirmed the diagnosis, showing widespread edema and late gadolinium enhancement consistent with severe acute myocarditis. The patient was managed conservatively regarding the bacterial infection but received aggressive cardiac support. Treatment included anti-inflammatory therapy with colchicine and ibuprofen, alongside the initiation of guideline-directed medical therapy for heart failure, including bisoprolol, dapagliflozin, eplerenone, and ramipril. The patient showed rapid clinical improvement and was discharged after six days. Follow-up imaging at two months demonstrated normalization of cardiac function with an LVEF of 58%, and cMRI at three months confirmed resolution of the acute inflammation. This case highlights the necessity of considering myocarditis in young patients presenting with chest pain following gastrointestinal infections. It further illustrates the potential benefit of early initiation of the "four pillars" of heart failure therapy in recovering myocardial function in bacterial myocarditis.

## Introduction

Myocarditis, the inflammation of the myocardium, presents with a constellation of symptoms and can lead to severe heart failure [1]. While viral infections are the leading cause of acquired myocarditis, other etiologies include immune disorders, toxins, and bacterial infections [2]. A potential bacterial agent is *Campylobacter jejuni* (*C. jejuni*), a common cause of gastroenteritis worldwide [3].

*Campylobacter* species gastroenteritis is typically self-limiting; however, it is associated with severe extraintestinal complications such as colitis, reactive arthritis, Guillain-Barré syndrome, and Miller-Fisher syndrome [3]. Myocarditis, though a lesser-known complication, carries significant morbidity. We report a case of severe acute myocarditis following a confirmed *C. jejuni* infection in a young adult, presenting with a significant reduction in left ventricular function.

## Case presentation

A previously healthy 19-year-old male patient presented to the emergency department (ED) with sudden-onset severe central chest pain. He described the pain as a tightening sensation, radiating to his neck and left arm, associated with paresthesia and diaphoresis. Three days prior to admission, he developed symptoms of gastroenteritis, specifically muscle aches, vomiting, and non-bloody watery diarrhoea. He had no past medical or family history of cardiac disease. He reported occasional vaping and alcohol use. There was no recent travel or identifiable food trigger.

On arrival, his observations included a temperature of 38.7°C, blood pressure of 93/53 mmHg, pulse rate of 77 beats per minute, and respiratory rate of 18 breaths per minute, with oxygen saturation of 96% on room air. Cardiovascular examination revealed normal heart sounds, and chest auscultation was clear. Abdominal examination demonstrated a soft, non-tender abdomen.

**Figure 1:**
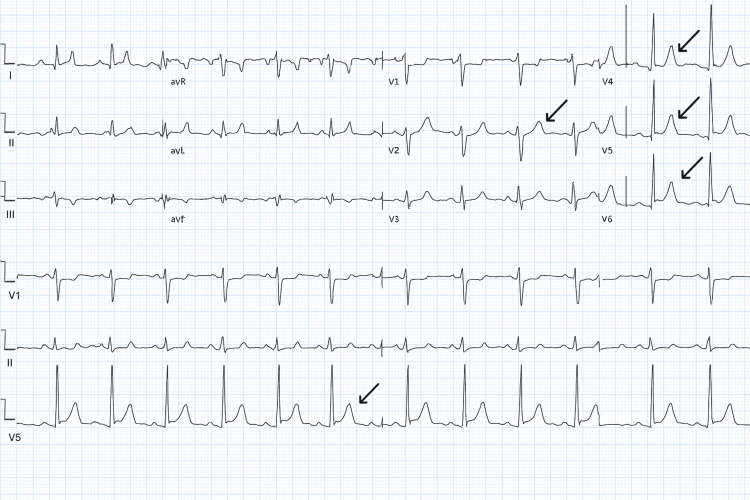
ECG demonstrating diffuse non-specific ST-segment elevation; arrows demonstrating ST elevation in V4,V5,V6

Initial studies, including the electrocardiogram (ECG), showed a normal sinus rhythm with non-specific ST-segment changes (Figure 1). Chest radiography demonstrated normal cardiomediastinal contours and no focal lung abnormalities.

**Figure 2:**
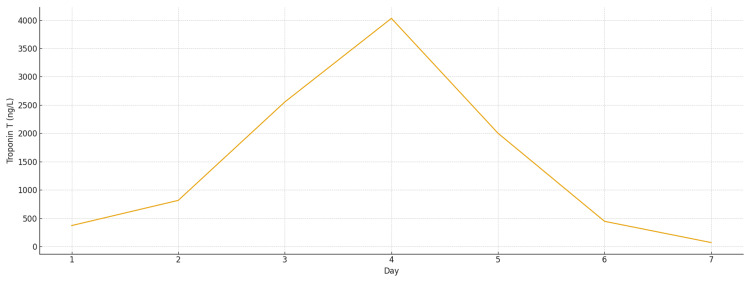
Rise and fall of troponin T in acute myocardial inflammation

Laboratory investigations showed a normal full blood count and renal function. C-reactive protein was elevated at 146 mg/L, and the initial troponin on admission measured 371 ng/L, rising to a peak of 4030 ng/L before normalising by the time of discharge (Figure 2; Table 1). After three days of admission, stool polymerase chain reaction (PCR) detected a *Campylobacter *species, while additional blood tests, including an autoimmune screen and *Borrelia burgdorferi* serology, were negative.

**Table 1:** Trend of troponin levels throughout admission (normal reference range: <13 ng/L)

Day	Troponin T (ng/L)
Day 1	371
Day 2	816
Day 3	2554
Day 4	4030
Day 5	2002
Day 6	446
Day 7	71

An echocardiogram performed on admission showed mild left ventricular dilatation with globally impaired systolic function (left ventricular ejection fraction (LVEF) 43%) and diffuse hypokinesis. Right ventricular function was also reduced, although chamber size remained normal, and the left atrium was mildly dilated.

**Figure 3:**
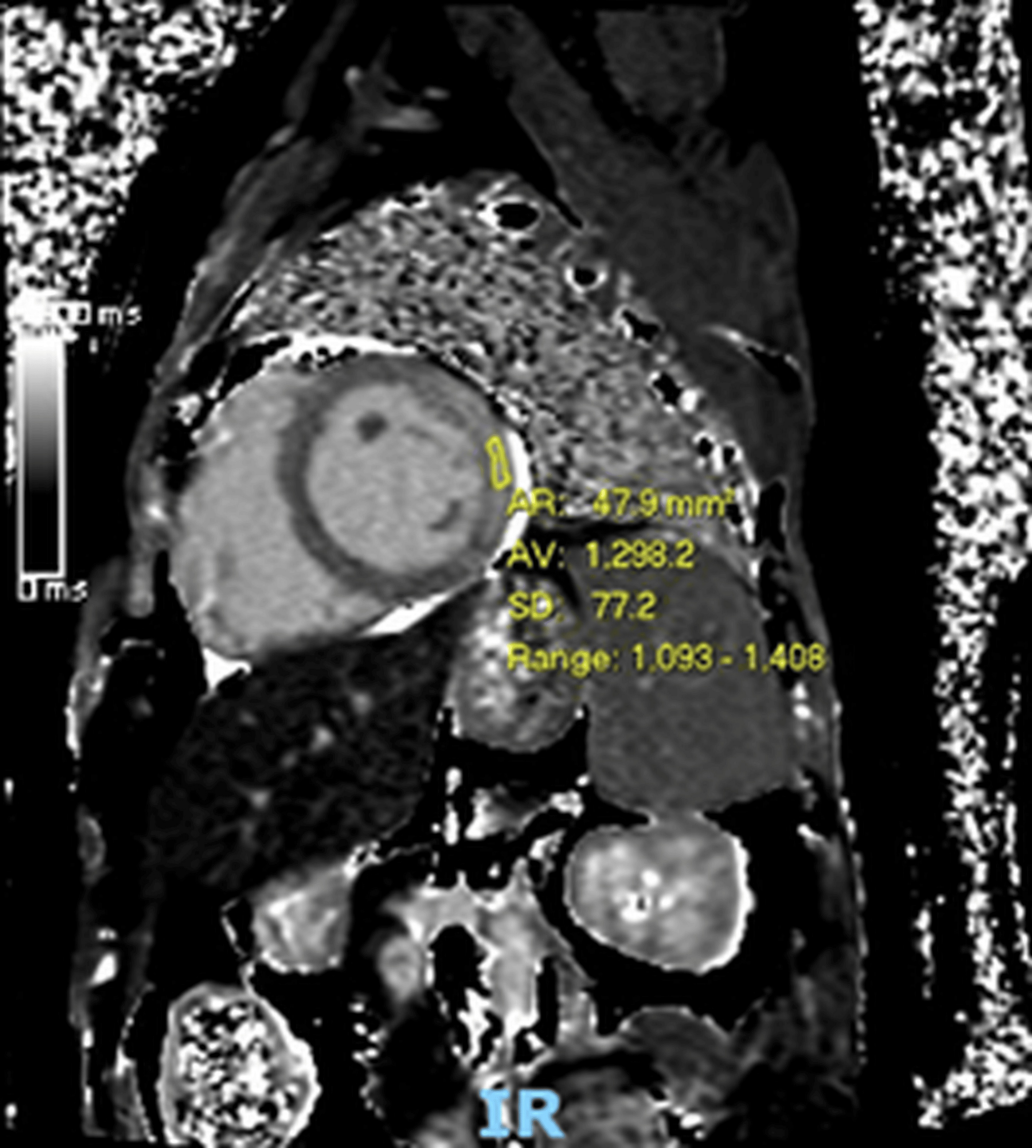
Cardiac MRI demonstrating myocardial oedema and late gadolinium enhancement T2 mapping images show markedly elevated T2 relaxation times, consistent with increased myocardial water content and active inflammation. The short-axis view demonstrates extensive mid-wall and epicardial late gadolinium enhancement involving the lateral wall from base to apex, in keeping with acute myocarditis under the Lake Louise Criteria.

Cardiac magnetic resonance imaging (MRI) demonstrated widespread myocardial oedema with extensive mid-wall and epicardial late gadolinium enhancement involving the lateral and anterior walls, associated with regional hypokinesis. The LVEF on cardiac MRI was 51% (Figure 3). Taken together, these findings were consistent with severe acute myocarditis.

Given the presentation and early imaging findings, the patient was started on ibuprofen 400 mg three times daily, which was subsequently switched to colchicine 500 micrograms twice daily for a planned three-month course. In view of his reduced left ventricular systolic function, guideline-directed heart failure therapy was initiated, including bisoprolol 2.5 mg once daily, dapagliflozin 10 mg once daily, eplerenone 25 mg once daily, and ramipril 5 mg once daily. These medications were introduced at low doses and titrated as tolerated.

His chest pain resolved during admission, and he remained clinically stable. He was discharged on day seven with plans for outpatient follow-up, including repeat echocardiography and 24-hour Holter monitoring. His case was subsequently reviewed at the multi-disciplinary team meeting to guide ongoing imaging and follow-up strategy.

**Figure 4:**
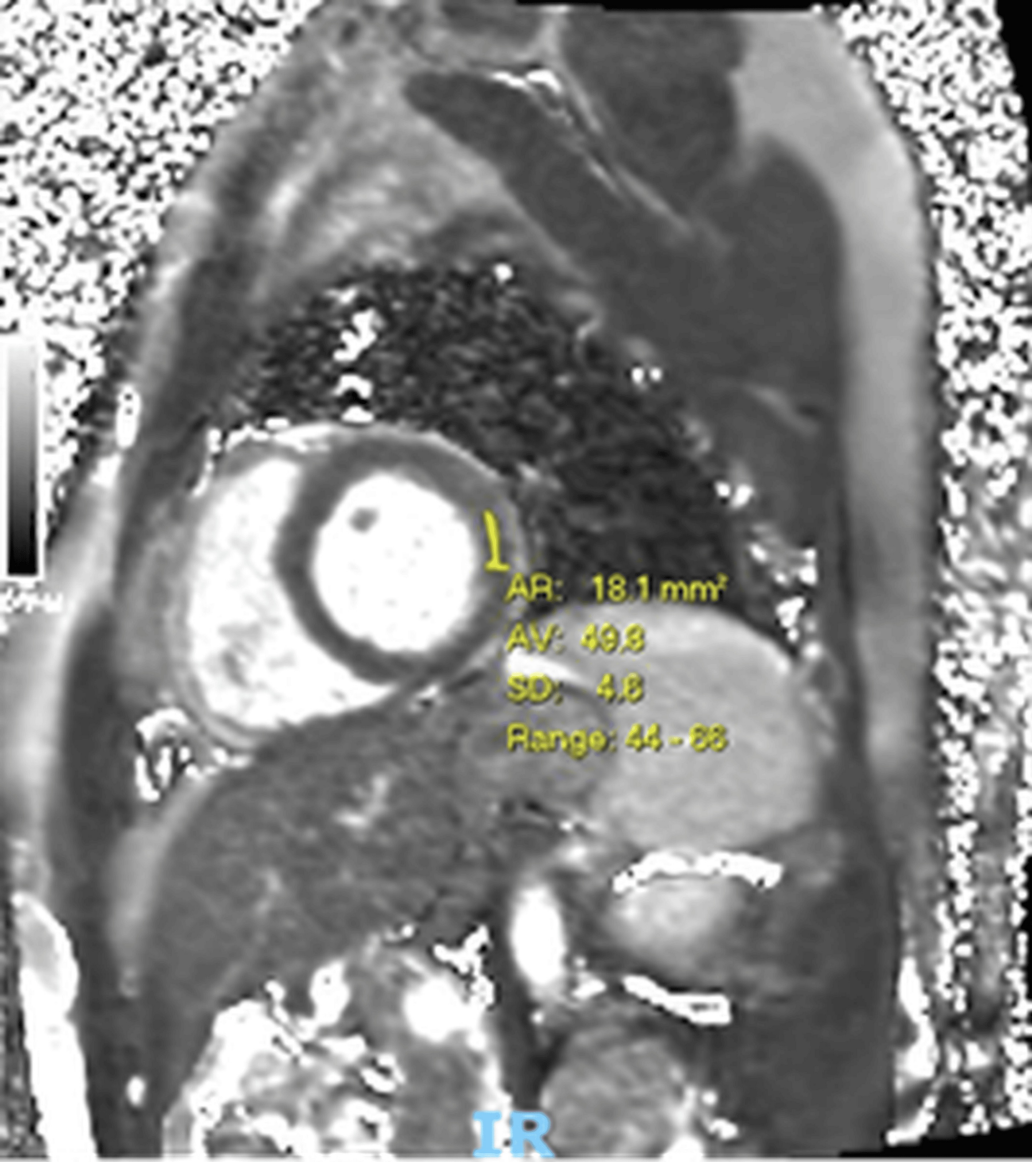
Follow-up cardiac MRI demonstrating resolution of myocardial oedema Three months after discharge, cardiac MRI T2 mapping demonstrates normalized or near-normalized myocardial T2 values (average: 49.9 ms; reference range: 44–68 ms). The restoration of T2 values to the normal range indicates resolution of myocardial oedema, consistent with recovery from the acute inflammatory phase of myocarditis.

A follow-up echocardiogram demonstrated improved left ventricular systolic function with an ejection fraction of 58%, though the inferoseptal segment remained mildly dyskinetic. The 24-hour Holter monitor showed no significant arrhythmias and no evidence of heart block. Further improvement was observed on cardiac MRI performed three months later (Figure 4), with near-normalisation of function. A small area of residual mid-wall and epicardial lateral wall scar was noted and was not expected to regress further.

The patient was advised to avoid strenuous exercise for three months following completion of therapy and subsequently resumed normal activity without limitation. At a one-year follow-up appointment, he remained clinically well with no evidence of recurrent myocarditis or heart failure. He reported intermittent non-specific chest discomfort, which was fully evaluated and determined to be non-cardiac in origin.

## Discussion

Myocarditis remains a difficult diagnosis, as it often presents without specific or pathognomonic clinical features. Viral infections, including hepatitis B, adenovirus, and coxsackievirus, are the most frequently reported causes [3]. However, several enteric bacterial pathogens, such as *Shigella*, *Salmonella*, and *C. jejuni*, may also precipitate myocarditis and are among the leading global causes of gastrointestinal infection [4].

Although *C. jejuni*-associated myocarditis is rare, its incidence has been estimated at 16.1 per 100,000 person-years [5]. It appears to occur more frequently in otherwise healthy adolescent and young adult males [5]. The pathophysiology remains uncertain. Proposed mechanisms include direct bacterial invasion of myocardial tissue or inflammatory injury mediated by circulating bacterial toxins, rather than an autoimmune mechanism, given the short interval between gastrointestinal symptoms and cardiac involvement [6,7]. While *C. jejuni* produces multiple exotoxins, none are known to directly target cardiac tissue [6].

The variability in clinical severity between individuals exposed to the same pathogen remains incompletely understood. Genetic susceptibility is thought to play an important role [8]. Mutations affecting toll-like receptor pathways may increase susceptibility to viral myocarditis and cardiomyopathy [9]. Variants in interferon-induced transmembrane protein-3 have been associated with influenza-related myocarditis [10], and animal studies have demonstrated associations between autoimmune myocarditis and HLA-DQ8 expression [11]. Additional mechanisms, such as viral protease-mediated cleavage of structural myocardial proteins and dystrophin deficiency, may also contribute to disease development [12]. Kontorovich et al. demonstrated that patients with acute myocarditis displayed significantly higher rates of deleterious genetic variants (16.2%) compared with controls (7.2%), findings most pronounced in paediatric populations and in lymphocytic myocarditis [13]. These data reinforce the importance of genetic predisposition in modulating individual risk.

A structured clinical assessment remains essential for improving diagnostic accuracy. Basic investigations, including ECG, cardiac biomarkers, and inflammatory markers, provide valuable initial clues, while transthoracic echocardiography enables assessment of ventricular function and detection of complications. Cardiac MRI with gadolinium enhancement has become a central diagnostic tool, allowing non-invasive tissue characterisation based on the Lake Louise criteria and reducing reliance on endomyocardial biopsy [14]. Multi-modal imaging was of particular value in this case, demonstrating initial myocardial oedema with subsequent interval resolution following treatment.

A review of the literature identified 34 published adult case reports of *C. jejuni* myocarditis, underscoring the rarity of the condition and the lack of standardised treatment recommendations. Management is largely supportive, focusing on optimisation of haemodynamics, correction of electrolyte disturbances, and treatment of heart failure or arrhythmias when present. Antibiotics, including macrolides and fluoroquinolones, have been used, though there is no consensus on optimal regimen or duration [15,16]. In this case, anti-inflammatory therapy with nonsteroidal anti-inflammatory drugs (NSAIDs) and colchicine provided symptomatic improvement, while guideline-directed heart failure therapy was initiated to support ventricular recovery.

## Conclusions

Myocarditis remains a diagnostic challenge, and this case reinforces the importance of a thorough clinical history and examination to raise early suspicion and identify potential triggers. Prompt initiation of treatment, even before definitive investigations are completed, is essential when myocarditis is strongly suspected. Managing complications, including heart failure and potential arrhythmias, is critical to prevent long-term morbidity. As demonstrated in this patient, timely and appropriate therapy can lead to full recovery of cardiac function, highlighting the reversibility of myocarditis in many cases. Nevertheless, significant variability exists in how patients respond to the same infectious exposure, underscoring the need for further research into genetic and host factors that influence disease severity.
